# Bouveret Syndrome: A Rare Case of Gastric Outlet Obstruction Caused by an Impacted Gallstone

**DOI:** 10.7759/cureus.43893

**Published:** 2023-08-21

**Authors:** Shahzeb Saeed, Niloufar Maktabijahromi, Norhan Mohammed, Chukwuyem Ekhator, Mubashir Iqbal

**Affiliations:** 1 Internal Medicine, Army Medical College, Rawalpindi, PAK; 2 Family Medicine, St. George's University School of Medicine, St. George’s, GRD; 3 Pediatrics, St. George's University School of Medicine, St. George’s, GRD; 4 Neuro-Oncology, New York Institute of Technology College of Osteopathic Medicine, Old Westbury, USA; 5 General Practice, Allama Iqbal Medical College, Lahore, PAK

**Keywords:** gallstones, gallbladder diseases, duodenal obstruction, gastric outlet obstruction, gallstone ileus, bouveret's syndrome

## Abstract

Bouveret syndrome is a rare condition where a gallstone obstructs the gastric outlet. This report discusses its diagnosis, management, and the need for further research. Prompt recognition is crucial to prevent complications. CT scan with oral contrast aids in diagnosis. Treatment options include conservative measures, endoscopy, or surgery. A multidisciplinary approach is essential for successful management. More research is needed to understand this condition fully.

## Introduction

Bouveret syndrome, an exceptionally rare and unusual form of gallstone ileus, accounts for less than 1% of mechanical intestinal obstructions. Named after its first describer, Léon Bouveret, in 1896, this condition involves the migration of a large gallstone into the duodenum or pylorus, leading to gastric outlet obstruction. Despite its historical recognition, only a few hundred cases have been reported, underscoring its rarity and diagnostic complexities [1].

The pathogenesis of Bouveret syndrome unfolds in a sequence of events. In patients with cholelithiasis, a gallstone is believed to erode through the gallbladder wall, creating a cholecystoduodenal fistula. Subsequently, the gallstone may pass through the fistula and lodge in the duodenum or pylorus, causing gastric outlet obstruction. The impacted stone acts as a mechanical barrier, obstructing the passage of food and gastric contents [2].

The clinical presentation of Bouveret syndrome is variable and non-specific, posing diagnostic challenges. Patients often manifest symptoms resembling gastric outlet obstruction, such as abdominal pain, nausea, vomiting, early satiety, and weight loss. These symptoms can mimic other common causes of obstruction, such as peptic ulcer disease or malignancy. Consequently, diagnosing Bouveret syndrome necessitates a high index of suspicion, especially in patients with a known history of cholelithiasis [3].

This case report contributes to the limited literature on Bouveret syndrome, elucidating its clinical presentation, diagnostic intricacies, and management strategies. By augmenting our comprehension of this rare gastrointestinal disease, healthcare professionals can refine their diagnostic acumen, facilitate early intervention, and optimize patient outcomes. Collaborative research efforts are needed to expand the literature and establish evidence-based guidelines for Bouveret syndrome management.

## Case presentation

A 68-year-old male presented at the emergency department with worsening abdominal pain, nausea, vomiting, and inability to eat for five days. The pain was constant and centered in the epigastric region, intensifying after meals. He had no fever, melena, or hematemesis. The patient had a medical history of hypertension, hyperlipidemia, and cholelithiasis, but had not experienced biliary colic before.

During the physical examination, the patient seemed uncomfortable and slightly distressed. His vital signs were normal, with a blood pressure of 128/78 mmHg, a heart rate of 88 beats per minute, a respiratory rate of 16 breaths per minute, and an oxygen saturation of 98% on room air. The abdominal examination showed moderate distention with visible peristaltic waves, tenderness in the epigastric region upon palpation, and hypoactive bowel sounds. There were no signs of peritonitis, such as guarding or rebound tenderness.

**Figure 1:**
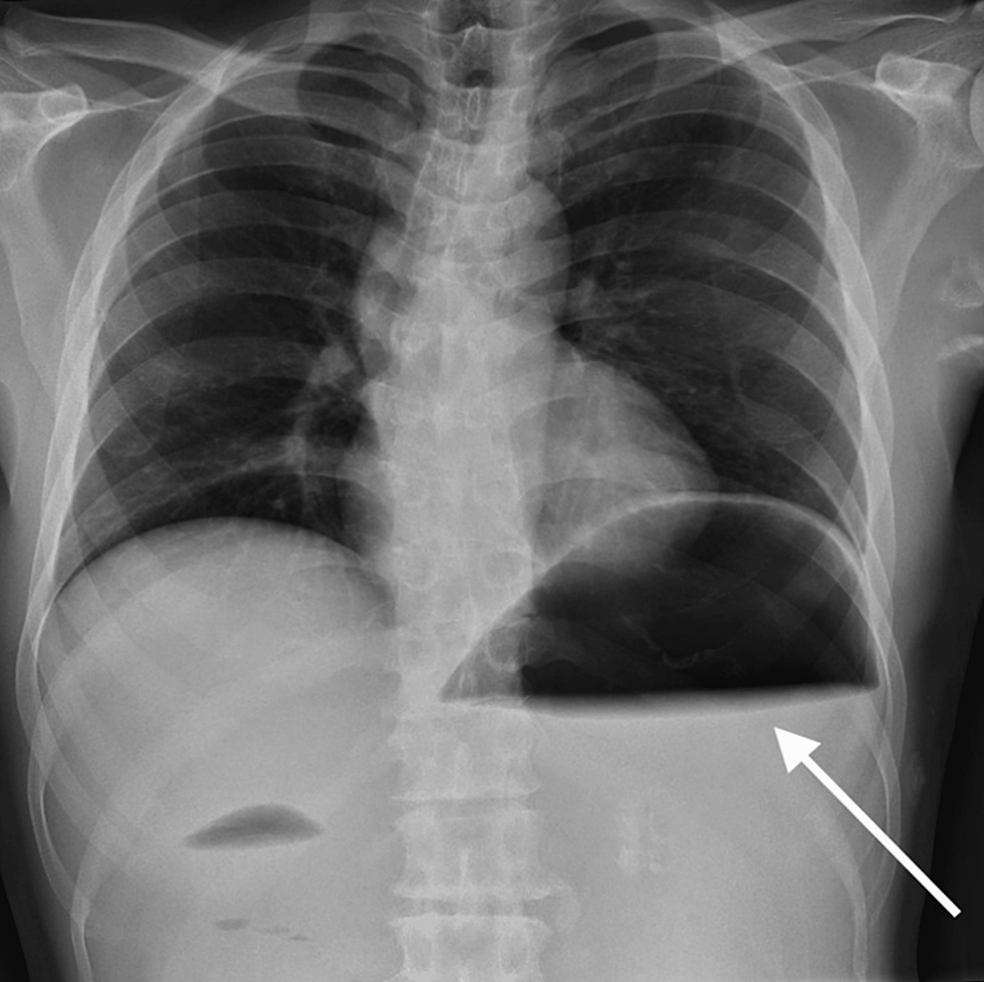
Marked gastric dilatation with an air-fluid level (arrow)

Initial laboratory tests including complete blood count, liver function tests, and serum amylase levels were within normal ranges except for white cell count, which was mildly raised. The electrolyte panel showed no abnormalities. The complete blood workup results are provided in Table 1. Radiograph displaying significant gastric dilatation with air-fluid levels, indicating gastric outlet obstruction (Figure 1).

**Table 1:** Complete blood workup MCHC: mean corpuscular hemoglobin concentration, ALT: alanine transaminase, AST: aspartate aminotransferase, ALP: alkaline phosphatase; INR: international normalized ratio

Coagulation Profile		Reference range
Prothrombin Time	12	Up to 13 seconds
INR	1.0	0.9-1.3
Control Time	26	25-35 seconds
Activated Partial Thromboplastin Time	28	Up to 31 seconds
Hemogram		
WBC count	13.6	4-11 x10^9^/L
Total RBC	3.8	3.8-5.2 x10^12^/l
Hemoglobin	13.7	13-18 (g/dL)
Hematocrit	36	35-46%
Mean Corpuscular Volume	78	77-95 fl
Mean Corpuscular Hemoglobin	33	26-32 (pg)
MCHC	35	32-36 (g/dL)
Platelets	412	150-400 x10^9^/L
Neutrophils	81	40-80%
Lymphocytes	20	20-40%
Monocytes	5	2-10%
Eosinophils	1.2	1-6%
Renal Function Tests		
Urea	48	10-50 mg/dl
Serum Creatinine	0.7	0.5-0.9 mg/dl
Liver Function Tests		
Bilirubin total	1.7	0.3-1.2 mg/dl
Total protein	5.6	5.7-8.2 g/dl
Albumin	3.5	3.2-4.8 g/dl
ALT	56	Up to 40 U/L
AST	36	Up to 40 U/L
ALP	321	40-120 U/L
Serum Electrolytes		
Sodium	136	135-145 mmol/L
Potassium	4.7	3.5-5 mmol/L
Chloride	100	98-107 mmol/L
Calcium	8.4	8.5-10.5 mg/dl

**Figure 2:**
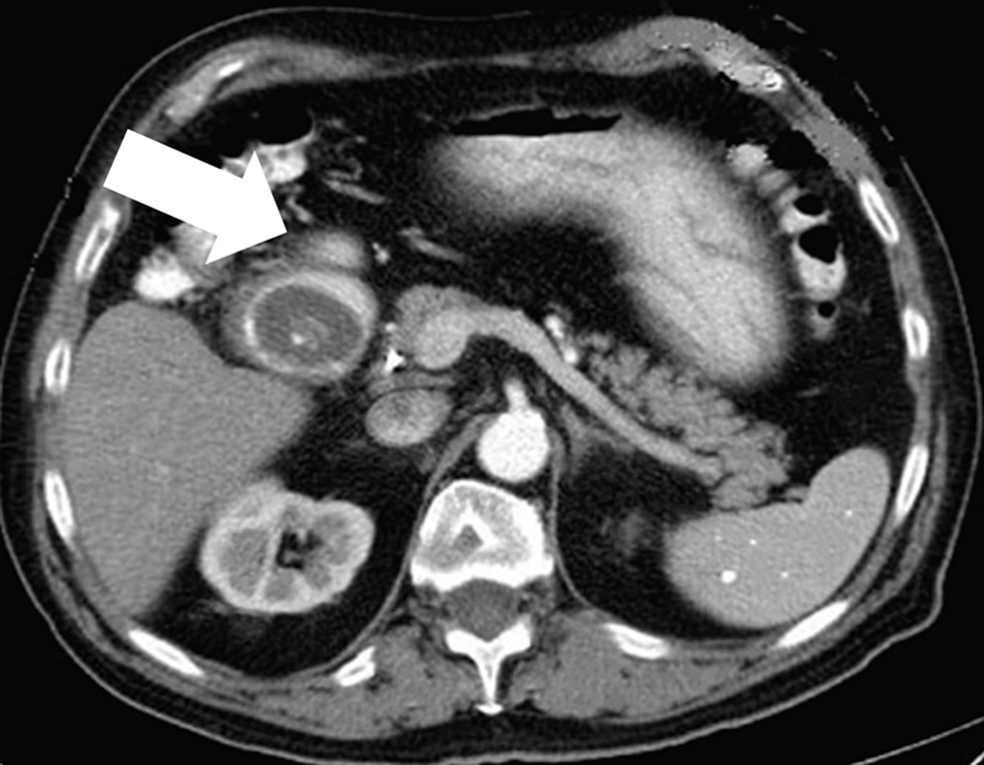
A large gallstone measuring approximately 3 cm in diameter (arrow) impacted in the duodenal bulb

The patient was promptly admitted for further investigations due to clinical presentation and imaging findings, and a CT scan with oral contrast was performed. The CT scan revealed a 3 cm gallstone lodged in the duodenal bulb, causing gastric outlet obstruction (Figure 2). Moreover, a cholecystoduodenal fistula was identified during the investigations. No associated complications were noted.

Bouveret syndrome was diagnosed based on the clinical symptoms and imaging results. A multidisciplinary team, including general surgery, gastroenterology, and interventional radiology, collaborated on the management plan. The patient and his family were informed about the various treatment options, including their risks, benefits, and potential complications.

Despite attempting conservative management, the patient's symptoms did not improve, and gastric decompression showed no progress over 48 hours. Given the gallstone's large size and impacted location, endoscopic retrieval was considered high-risk, leading to the decision for surgical intervention. Following informed consent, the patient underwent an exploratory laparotomy.

The surgical intervention necessitated a midline incision for an in-depth exploration of the abdominal cavity. Upon investigation, a gallstone, measuring approximately 3 cm in diameter, was discovered impacted in the duodenal bulb, confirming its role in the gastric outlet obstruction. A meticulously executed gastrotomy facilitated the gallstone's extraction, with rigorous care to prevent any spillage or contamination. Simultaneously, a cholecystoduodenal fistula was detected and repaired with an omental patch. The gastrotomy site was then securely closed using interrupted sutures. To preclude the recurrence of Bouveret syndrome, a concurrent cholecystectomy was carried out. The patient subsequently had an uneventful recovery, showing symptom resolution and no postoperative complications.

Post surgery, the patient was closely monitored in the surgical intensive care unit. Nasogastric decompression was maintained to ensure proper gastric decompression. Intravenous fluid resuscitation, electrolyte correction, and antibiotic therapy were continued. Over time, the patient's symptoms improved, with abdominal pain, nausea, and vomiting resolving. Oral intake was gradually reintroduced, starting with clear liquids and progressing to a regular diet as tolerated.

The patient's recovery after surgery was smooth, without any surgical site infection, wound dehiscence, or complications. Throughout the hospital stay, the patient remained hemodynamically stable. On the sixth postoperative day, the nasogastric tube was removed, and the patient continued to tolerate a regular diet without signs of recurrent gastric outlet obstruction. Follow-up imaging, including abdominal X-ray and CT scan, showed no residual stones or complications.

The patient was discharged on the seventh postoperative day with appropriate postoperative care instructions and a referral for outpatient follow-up. During follow-ups, the patient was thoroughly assessed with no remarkable or adverse findings noted. Additionally, potential complications related to the fistula repair, such as anastomotic leakage, wound infection, and bowel obstruction, were evaluated, but no significant abnormalities were identified. The patient reported a complete resolution of symptoms and expressed satisfaction with the surgical intervention. 

## Discussion

Bouveret syndrome is a rare and complex clinical condition characterized by gastric outlet obstruction resulting from a gallstone lodged in the duodenum or pylorus. Its diagnosis presents a challenge due to the non-specific clinical presentation, often resembling other common causes of gastric outlet obstruction. Effectively managing Bouveret syndrome requires a multidisciplinary approach, involving general surgery, gastroenterology, and interventional radiology. Treatment options encompass conservative measures, endoscopic techniques, and surgical intervention, with the chosen approach depending on patient stability, gallstone size and location, and the presence of associated complications [4]. Prompt recognition, precise diagnosis, and appropriate management are crucial in preventing complications and improving patient outcomes [5].

Imaging studies play a pivotal role in confirming the diagnosis of Bouveret syndrome. Abdominal radiography may reveal gastric dilatation with air-fluid levels, suggesting an obstructive pattern. However, the definitive diagnostic tool is a CT scan with oral contrast, providing detailed visualization of the impacted gallstone and its precise location within the duodenum or pylorus. The CT scan also aids in evaluating associated complications, such as gallstone-related perforation or inflammation [6].

In our case, the patient exhibited typical symptoms of gastric outlet obstruction, including abdominal pain, nausea, vomiting, and intolerance to oral intake. Abdominal radiography showed pronounced gastric dilatation with air-fluid levels, raising suspicion of gastric outlet obstruction. CT scan confirmed the diagnosis by revealing a large gallstone lodged in the duodenal bulb, causing significant luminal narrowing and gastric outlet obstruction.

Conservative management, including nasogastric decompression, fluid resuscitation, electrolyte correction, and antibiotics, may be attempted in stable patients without evidence of peritonitis. The aim of this approach is to alleviate gastric distention, correct electrolyte imbalances, and facilitate the spontaneous passage of the gallstone. However, it should be noted that conservative management may not be efficacious in cases involving large or impacted stones, as observed in our patient, necessitating further intervention to address persistent symptoms.

Endoscopic techniques such as endoscopic lithotripsy, mechanical lithotripsy, or laser lithotripsy have proven successful as minimally invasive approaches for gallstone retrieval in selected cases of Bouveret syndrome. Nonetheless, the feasibility and success rate of endoscopic interventions rely on factors such as stone size, location, and the expertise of the endoscopist. In our case, endoscopic retrieval was considered high risk due to the large size and impacted location of the gallstone, leading to the decision for surgical intervention [4].

Surgical intervention remains the definitive treatment for Bouveret syndrome, particularly when dealing with large or impacted gallstones. The surgical procedure typically involves an exploratory laparotomy, identification of the impacted stone, and extraction through a gastrotomy or duodenotomy. Primarily, closure of the defect is performed to minimize the risk of postoperative complications, such as bleeding or leakage. Concurrent cholecystectomy is often carried out to prevent recurrence, given the frequent association of cholecystoduodenal fistulae with Bouveret syndrome [4].

The prognosis of Bouveret syndrome depends on several factors, including the patient's overall health status, the presence of associated complications, and the promptness of intervention. Delayed diagnosis and treatment can lead to significant morbidity and mortality, such as gastric necrosis, perforation, or sepsis. Therefore, early recognition and intervention are crucial in achieving favorable outcomes [3].

Considering the rarity of Bouveret syndrome, limited literature exists regarding its optimal management strategies. Thus, the choice of treatment should be individualized based on the patient's clinical condition, gallstone size and location, and the expertise of the medical team. Further research, including case reports and collaborative studies, is necessary to build up the literature and establish evidence-based guidelines for the management of Bouveret syndrome.

## Conclusions

Bouveret syndrome is a rare gastrointestinal condition with symptoms of gastric outlet obstruction. Early recognition, accurate diagnosis, and prompt intervention are crucial for successful management. CT scan with oral contrast aids in diagnosis. Treatment options include conservative measures, endoscopy, or surgery, with the latter being definitive for large or impacted stones. Timely intervention and a multidisciplinary approach are essential for positive outcomes. More research and case reports are needed for optimal management.
